# Research Progress on Thermal Insulation Material Systems for High-Speed Aircrafts

**DOI:** 10.3390/ma19071311

**Published:** 2026-03-26

**Authors:** Xinke Jiang, Yongcai Guo, Yong Zhou

**Affiliations:** Key Laboratory of Optoelectronic Technology and System of Ministry of Education, College of Optoelectronic Engineering, Chongqing University, Chongqing 400044, China; 202408131164@stu.cqu.edu.cn

**Keywords:** high-speed aircraft, frictional drag, thermal insulation materials, high temperature resistance

## Abstract

During high-speed flight, intense friction on the aircraft surface always occurs due to atmospheric fluid medium. The resultant high frictional drag will trigger a significant aerothermal effect, and thus raise the surface temperature sharply to 1000–3000 °C. This extreme heat not only remarkably reduces the aerodynamic efficiency but probably also causes thermal failure of the structural integrity and damage of internal components. Therefore, robust heat-resistant materials are the preferred choice for designing high-speed aircraft due to their benign tolerance to high temperature, oxidation and ablation as well as large strength and durability. This work systematically unveils the generation mechanism of frictional drag in high-speed flight and introduces the characteristics and applications of typical thermal insulation materials (TIMs). After that, the recent progress in a thermally protected material system including metal-based alloys and metal-doped compound materials, ultra-high-temperature ceramics (UHTCs), carbon (C)/carbon (C) and C/SiC composites, ceramic matrix composites (CMCs), UHTCs-modified C/C and C/SiC composites is conducted. Finally, the current technical bottlenecks are discussed, simultaneously proposing the development direction of novel TIMs for the potential applications for high-speed aircrafts.

## 1. Introduction

With ever-increasing development of aerospace technology in the 1960s, there have been explosive breakthroughs in the technology of high-speed aircrafts with intensive research enthusiasm. Currently, the maximum flight speed through rocket boost or gliding technology has exceeded Mach 20, while the general stable flight speed can reach Mach 5–10 [1,2,3,4], as displayed in Table 1.

**Figure 1:**
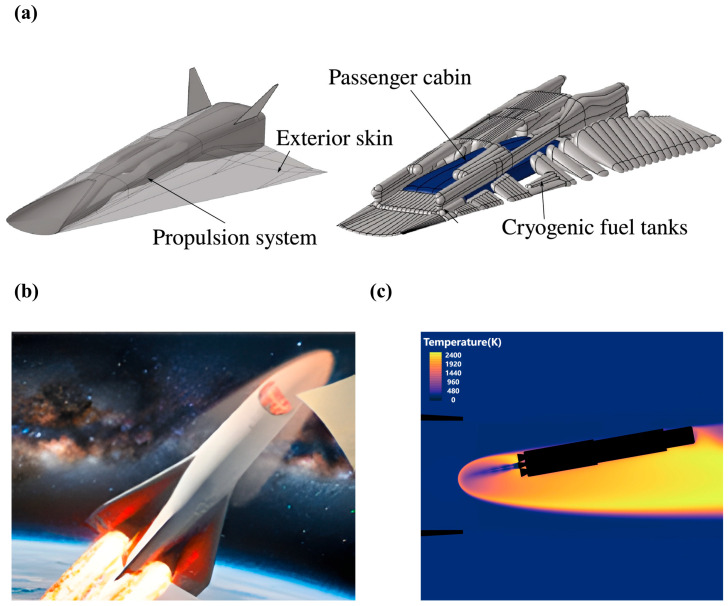
Surface temperature and surface ablation of high-speed aircraft: (**a**) the aerodynamic layout and fuel tank design of the STRATOFLY MR3 rocket [4], (**b**) aircraft surface ablation [5], and (**c**) high temperatures resulting from post-combustion, as determined by CFD simulations [6].

Meanwhile, as the flight speed increases, the aerodynamic heating effect becomes significant. Accompanied by the conversion of a substantial amount of kinetic energy into thermal one, intense air friction is produced on the vehicle surface. Figure 1a,b schematically show the “STRATOFLY” project aircraft, which consists of a wave lift body and an integrated dorsal propulsion system. The phenomenon of surface ablation is always determined by thermal simulation via computational fluid dynamics (CFD) (Figure 1c) [4,5,6]. Then, the heat is transferred to the wall, which causes a continuous rise of the surface temperature on the aircraft. When flying above Mach 10, the surface temperature readily exceeds 1000 °C, while the temperature of its nose cone and sharp leading edge even reaches 2500 °C. At Mach 16, the surface temperature exceeds 2000 °C [7].

Accompanied with the rapid increase in the flight speed, the proportion of frictional drag in the total drag increases sharply to more than 50% [8]. In some complex environments (high altitude, high Mach, high angle-of-attack maneuvers and trans-atmospheric flights), the frictional drag can account for 90% or more. In terms of flight performance, the frictional drag hinders the speed increase and the extension of flight range, which greatly lowers the flight effectiveness. To counter this adverse impact, the structural strength of aircrafts must be enhanced, which undoubtedly increases the total weight and further intensifies the contradiction between energy consumption and flight performance [9]. During the long service of high-speed aircrafts, some components must simultaneously endure a series of extremely harsh environments including large heat flux density [10], high heat load [11], high-pressure airflow, high-energy particle scouring [12], oxidation effects [13], etc.

To mitigate the serious negative effect from the frictional drag, mechanical structure improvement and material optimization have been initially proposed. For instance, shock wave formation ahead of a hypersonic aircraft can be alleviated using a spike-based flow control device [14]. The second approach is reverse jet technology, wherein a Mach disk forms in the reverse jet flow to establish pressure equilibrium between the separated shock wave and the jet, followed by the interaction with the oncoming flow to alleviate aerodynamic drag and surface heat load [15]. The third category comprises energy-based flow control devices [16], such as laser- or microwave-induced energy deposition. These devices introduce a localized high-energy region into the undisturbed oncoming flow and results in a substantial localized temperature rise within this region. Consequently, the kinetic energy and total pressure of the downstream flow are highly reduced, and the bow shock ahead of the aircraft is markedly weakened—or fully suppressed under optimal conditions [17].

In terms of TIMs, an insight into the material system can optimize and upgrade the thermal protection systems (TPSs) for active or passive aircraft [18,19], improve the aerodynamic performance, extend the structural life, and lay the foundation for higher speed and longer range. Meanwhile, high-performance TIMs favor the reduction in the accumulation and the conduction of frictional heat at the interface, which then effectively minimizes the fuel consumption and carbon emissions, and sustains a sustainable development.

Based on these facts, this work is dedicated to clarifying the coupling relationship between the frictional drag and thermal response of related materials, summarizing the recent progress of TIMs for high-speed aircrafts, and stressing the collaborative design strategies of multi-material systems.

**Figure 2:**
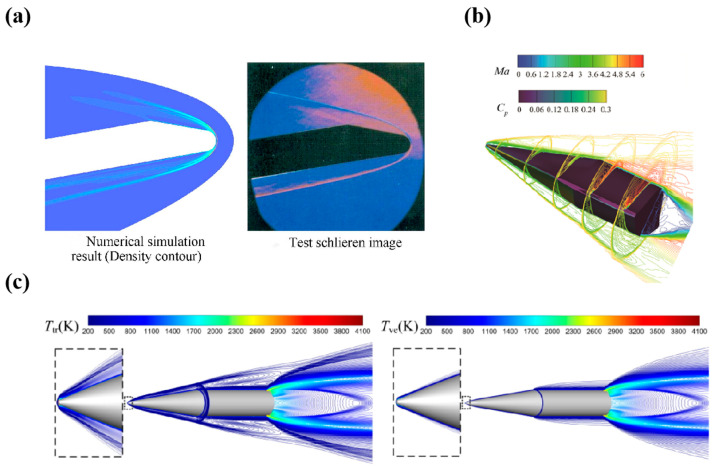
The distribution features of flow field on the model surface: (**a**) flow field at the symmetrical plane of the model [21], (**b**) visualization of surface pressure distributions and Mach number contours across different morphing positions [22], and (**c**) the variation in trans-rotational and vibro-electronic temperatures of the gas [23].

In the laminar or turbulent boundary layer on the aircraft surface due to high-speed airflow, the velocity gradient near the wall produces viscous shear stress as $\tau_{\omega }=\mu \left(\frac{\partial u}{\partial y}\right)y=0$ [20], where *u* and *μ* separately represent the flow velocity and the dynamic viscosity. The viscous shear action within the boundary layer sharply raises the temperature on the material surface. Figure 2a presents the flow field distribution on the symmetry plane of the aircraft model. The boundary layer structure is clearly resolved. Figure 2b presents the surface pressure distribution and Mach number contour lines at different deformation positions. Figure 2c shows the distribution of the cross-rotational temperature and the vibration electron temperature on the symmetry plane when the aircraft is flying at an altitude of 25 km and Mach 10. The evolution of gas viscosity coefficient follows the Sutherland formula μ∝T32, indicating an increase in wall shear stress with surface temperature. This effect alters the physical state of the material surface and the structure of the laminar flow field at the boundary. Also, it will affect the generation of frictional resistance, eventually forming a complex bidirectional coupling mechanism.

Moreover, the aircraft’s surface area and the thermophysical properties of its outer-surface material significantly influence the generation of frictional heating. In particular, the radiation coefficient and thermal conductivity (TC) of the surface material directly determine the operational stability of internal precision components. Provided that TIMs possess poor resistance against high temperature and ablation, rapid oxidation readily occurs in high-temperature environments. Then, these materials burn rapidly, and cause severe damage to its internal components [24]. Sun et al. [25] established an unsteady numerical method to investigate the characteristics of laser ablation and flow field for C/C materials under high-speed gas flow. The results find that the gas flow dynamics substantially influence the wall temperature at different locations. The total ablation rate at Mach 6 is 5.3% greater than that under quiescent conditions. The interaction between ablation injection and the high-speed freestream alters local flow characteristics, and establishes a transient high-pressure region that modulates wall pressure at different wall positions. These localized pressure variations substantially impact the sublimation rate of the wall, thereby influencing the overall ablation rate. Some oxidation reactions (carbon-based materials generate CO_2_, while silicon-based materials form SiO_2_ glass films) on the material surface also increase the surface roughness, and then the turbulent frictional drag. In addition, the thermal effect could induce deformation and phase transformation. The thermal expansion of the material at high temperature changes the surface curvature, and disrupts the smoothness. When exceeding the critical oxidation temperature, the weight loss on the surface could reach 0.05 mm/h, simultaneously producing pit-like defects of a matched scale with the boundary-layer thickness (≈100 μm for 50 μm scale defects at Mach 8), which directly affects the resistance characteristics.

In virtue of these facts, this work first introduces the aerothermal effect of high-speed aircraft, analyzes the serious harm to flight missions, and unveils the generation mechanism of frictional drag on the aircraft surface. Then, the key function of some TIMs and their representative examples are emphasized. Finally, the latest progress of various TIMs is summarized with the focus on their future development for high-speed aircrafts.

## 2. TIMs for High-Speed Aircrafts

**Figure 3:**
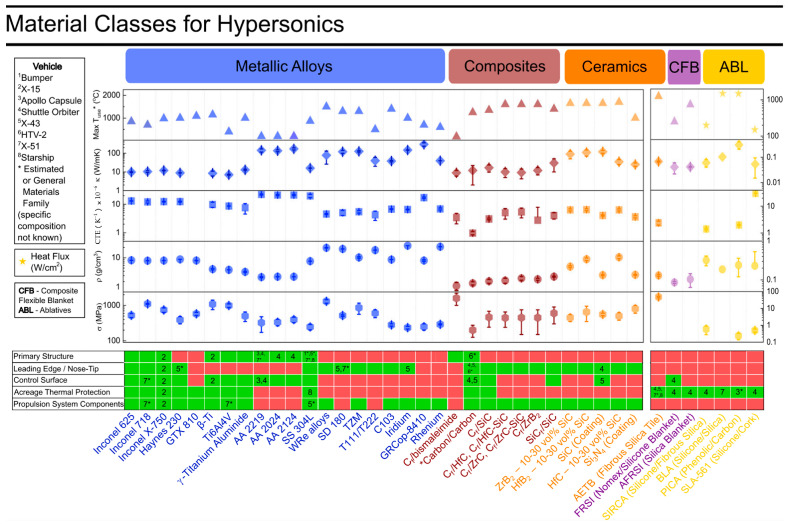
Development of thermal insulation material systems (* Estimated or general materials family (specific composition not known)) [26].

TIMs are the carrier of high-speed aircraft and the fundamental guarantee for breaking through the thermal barrier and achieving high-Mach flight. Every major improvement in the energy performance of an aircraft requires an update of TIMs as a prerequisite. Researchers have gradually developed light alloys, superalloys, refractory metals and their alloys, C/SiC composites, UHTC antioxidant-modified C/C and C/SiC composites, UHTC and carbon fiber (CF)-toughened C/UHTC composites, and C/C heat-resistant material systems (Figure 3). Existing heat protection systems have shown non-ablation within a broad range of 1600–2500 °C and moderate ablation at an extremely high temperature of 3000 °C [26]. Meanwhile, the interrelationships among different TIM systems have promoted many composite material systems. For instance, ceramic materials (e.g., SiC, ZrC) were introduced into C/C composites, which enhances the resistance to ablation and oxidation while maintaining a low density and a high-temperature resistance.

### 2.1. Metal-Based Alloys and Metal-Doped Compound Materials

The research and applications of metal-based superalloys within extremely high-temperature environments began earliest. There are outstanding achievements and wide applications in high-speed aerospace, high-temperature gas turbines, and industrial nuclear reactor equipment. Based on their resistance to high temperature, metallic materials can be roughly classified into high-temperature metals/alloys and refractory metals (melting point above 2200 °C) [27].

High-temperature metals/alloys typically operate above 600 °C, including Ti, Y, Ni, Co, etc. Among these, Ni-based superalloys gain the most popularity [28] owing to a maximum operating temperature of about 1200 °C. The merits of high toughness, easy processing, and thermal shock resistance enable Ni-based superalloys widely employed as the hot-section components of gas turbines and aerospace engines. With respect to traditional alloys, the oxidation resistance is markedly ameliorated by incorporating small and controlled amounts of Al (6 at%) [29,30], Cr (8 wt%) [31], or Si elements (15 wt%) [32] by forming a stable and dense surficial oxide layer at high temperatures.

**Figure 4:**
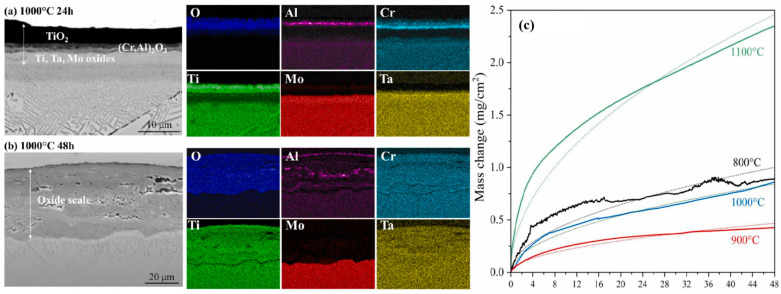
The cross-sectional SEM images of 48Ti–25Mo–12Ta–10Cr–5Al alloy after oxidation at 1000 °C in synthetic air and related EDS mapping for (**a**) 24 h and (**b**) 48 h, and (**c**) the mass change during the oxidation process at 800–1100 °C [38].

Refractory metals and their alloys serve as critical structural materials due to remarkably high melting points [33], excellent strength and hardness retention at elevated temperatures, and outstanding resistance to ablation and corrosion, particularly in extreme thermal environments encountered at leading edges, combustion chambers, and nose tips of hypersonic vehicles. Metals with a high melting point, including W (3414 °C), Mo (2622 °C), Re (3180 °C), and Ta (2996 °C) [34], can be widely used in various extreme environments. In a previous work [35], Pan et al. utilized W-doped Zr-based alloys, which raised the melting point to 1138 °C and improved overall properties of the obtained superalloy (elastic modulus, elastic anisotropy, thermodynamic properties, etc.). In addition, the melting point of Co-based alloys could be raised by approximately 200 °C by adding Re [36]. Also, the compressive strength of W matrix/W-Ti-Fe core superalloy could maintain 500 MPa at 1000 °C [37]. Figure 4 shows the intermittent oxidation test of Ti–Mo–Ta–Cr–Al alloy at 1000 °C [38]. After oxidation for 24 h, a dense oxide layer is found (Figure 4a). This layer exhibits a laminated structure consisting of a solid solution of TiO_2_ and (Cr, Al)_2_O_3_. The elemental analysis reveals distinct concentration gradients of Al and Cr, with Al being significantly enriched at the surface. Beneath this outer layer, a secondary oxide layer rich in Ti, Ta, and Mo appears. On the contrary, following another oxidation for 48 h, a thicker and porous oxide layer is produced, which is primarily composed of Ti, Cr, and Ta and scattered regions of Al-rich oxides embedded within the intermediate zone (Figure 4b). The EDS analysis confirms that Mo is absent in the oxide film after this extended exposure. As illustrated in Figure 4c, the weight gain of each alloy does not increase monotonically with the oxidation temperature. This anomalous behavior arises from the volatilization of MoO_3_, which intensifies with the Mo content.

Most metallic materials exhibit poor oxidation resistance and tend to form non-protective, volatile oxide layers [39] as the oxidation kinetics are accelerated at high temperature. This behavior accelerates the growth of the oxide layer, and reduces the creep and fatigue resistance, eventually threatening the reliability and service life. To this end, high-entropy alloys (HEAs), which are single-phase, random solid-solution materials containing five or more elements with near-equimolar atomic proportions, have been proposed to suppress the formation of intermetallic compounds and thermodynamically stabilize the single-phase solid solution. Thanks to sluggish diffusion kinetics, the bonding between metal cations and oxygen ions is retarded while reactions between volatile oxides (e.g., MoO_3_, V_2_O_5_) and simple oxides (e.g., Al_2_O_3_, SiO_2_) are promoted, thereby facilitating the in situ formation of the protective complex oxide scales. Moreover, the design strategies for HEAs substantially expand the space for compositional design, enabling systematic exploration of novel oxidation-resistant material systems [40].

In addition, HEAs exhibit exceptional hardness, outstanding resistance to wear, elevated-temperature deformation, and environmental degradation (oxidation and hot corrosion), and benign low-temperature ductility and superplasticity [41]. HEAs can be classified into iron group metals and similar transition metals, transition refractory metals, platinum group elements, gold, light elements and lanthanide alloys. When it comes to the oxidation resistance, a series of Hf-Nb-Ta-Ti-Zr alloys through vacuum arc melting are prepared [42]. By using the powder-pack aluminizing process and subjecting the alloy to aluminization treatment with Al powder (45 wt% Al, 45 wt% Al_2_O_3_, 10 wt% NH_4_Cl), a protective oxide film is formed below 1000 °C, thereby enhancing the oxidation resistance. Nevertheless, this antioxidant layer deteriorates markedly at 1300 °C due to reduced density and diminished continuity of the oxide scale, which facilitates inward oxygen diffusion and consequently compromises the oxidation resistance. Table 2 presents a comparison of typical material parameters for each system of metallic materials.

**Figure 5:**
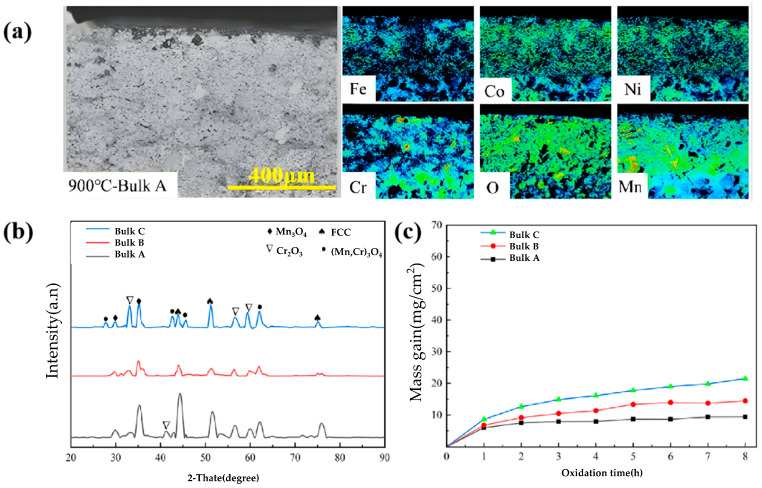
The morphology and oxidation kinetics curves of porous FeCoNiMnCr_x_ alloy at 900 °C: (**a**) EPMA image, (**b**) XRD pattern, and (**c**) oxidation kinetics curve [44].

HEAs based on light and lanthanide elements are prone to complete oxidization. At a pressure close to an atmospheric one, platinum group metals (such as Ru) can deteriorate the oxidation resistance, so their oxidation phenomena at high temperature are hardly worth studying [43]. HEAs based on iron group and transition refractory metals exhibit fewer compositional limitations than traditional ones (stainless steel, Ni-based alloys, etc.), and accommodate higher concentrations of alloying elements including Al, Cr, and Si, without precipitating brittle intermetallic phases. These elements promote the oxidation of protective oxides at the alloy surface, thereby establishing a continuous, adherent, and diffusion-limiting oxide scale that significantly enhances the corrosion endurance at high temperature. Among HEAs based on iron group metals, Al-Co-Cr-Fe-Ni and Mn-Co-Cr-Fe-Ni systems gain the widest popularity. As Al and Cr in their compositions are more prone to oxidation than other components in these alloys, their Pourbaix ratios are greater than 1, which enables the derived oxide films to be protective. In addition, such HEAs show low elemental diffusivity, and it is difficult to form oxide particles inside (Figure 5). Based on the electron probe micro analyzer (EPMA) image at 900 °C (Figure 5a), Cr- and Mn-rich oxide layers are produced on the cross-section [44]. Meanwhile, the concentration of O on the surface increases with a clear inner oxidation, which leads to the Ni depletion. As NiO material produced at high temperatures is unsaturated, it falls off continuously to create a new one, which further depletes Ni with time. Bulks A, B, and C are oxidized into Mn_3_O_4_, Cr_2_O_3_, and (Mn, Cr)_3_O_4_ at 900 °C, respectively (Figure 5b). The unstable Mn3O4 phase above 877 °C is produced under this situation (Figure 5c). At the same temperature, the sample shows increased oxidation gain with Cr content.

Butler et al. [45] studied the microstructure and oxidation behavior for a series of HEAs based on AlCoCrFeNi. It is found that the oxidation resistance can be intensified with the Al content by forming a continuous and adherent Al_2_O_3_ film. Similarly, three different samples of AlCoCrFeNi, AlCoCrCu_0.5_FeNi and AlCoCrCuFeNi are oxidized at 1000 °C within air for 100 and 500 h, respectively [46]. The addition of Cu improves the corrosion resistance while the increased content of Cu weakens the link between the oxide film and the material surface. The diffusion coefficient of the Mn-Co-Cr-Fe-Ni system is lower than that of traditional face-centered cubic lattice alloys. Within 1100 h oxidation experiments within air at 650 and 750 °C, the oxidation resistance of the Cr-containing HEA system is increased with the Cr content [47]. Thermodynamic calculations indicate that spinel-type MnCr_2_O_4_ serves as the alloy surface/oxide layer. Therefore, the alloys with low Mn and high Cr contents can be better applied in high-temperature oxidizing environments. In another report [48], Mn_19_Co_21_Cr_20_Fe_20_Ni_20_ alloy is oxidized. The oxidation species of Mn and Cr elements are produced rather than Fe, Co and Ni ones due to more negative Gibbs free energy for the former.

Despite the good resistance to heat and corrosion for refractory metals and their alloys, the high density limits their further application in weight-sensitive fields such as aerospace. In addition, poor oxidation resistance remains. Within high-temperature oxidizing environments, volatile and easily shedding oxides (WO_3_, MoO_3_, Ta_2_O_5_, etc.) are formed before reaching the service temperature, which significantly reduces the material strength and deteriorates the performance. Therefore, the application of refractory metals and related alloys in high-temperature environments is largely limited.

### 2.2. C/C and Its Composites

The C/C composites refer to CF-reinforced composites, which are made of CFs or their fabrics as the reinforcing phase while C or graphite forms the matrix through a series of densification and graphitization treatments [49]. Compared with metal alloys, the C/C composites showed a much lower density (0.18–2.2 g/cm^3^). In addition, there are other favorable properties such as a high resistance to temperature and ablation, large compressive strength of >80 MPa/(g/cm^3^) and modulus of >50 GPa/(g/cm^3^), excellent TC, low coefficient of thermal expansion (CTE, 0.6 × 10^−6^/°C–1.4 × 10^−6^/°C), and benign strength retention rate at high temperature. Most importantly, they maintain excellent specific strength and good fracture toughness at high temperature, and even exhibit increased strength with temperature (1000–2300 °C) [50]. During the re-entry process of hypersonic aircraft where the temperature at the tip exceeds 3000 °C [51], the C/C composites exclusively withstand this ablation environment because of low density and excellent mechanical retention. Thus, the composites are also the most widely used material for throat linings of solid propulsion rockets [52]. In addition, the composites possess unique resistance to high-temperature oxidation as well as severe scouring and ablation from high-pressure gas and high-speed particles.

**Figure 6:**
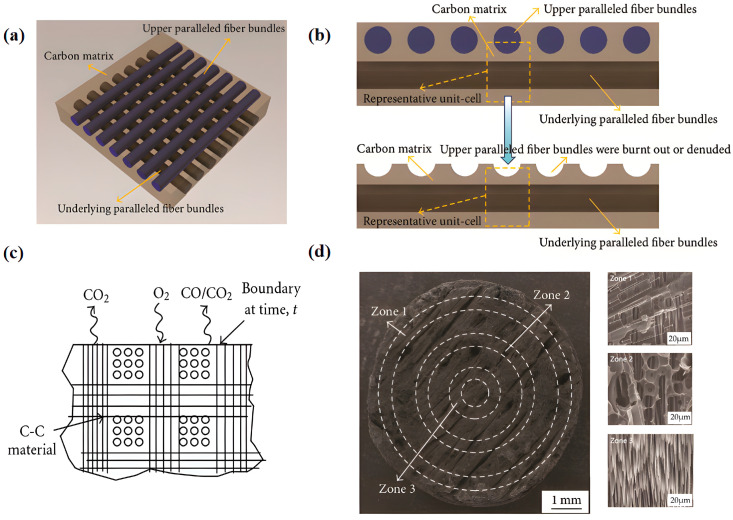
Structural and morphological characterization of the C/C composites and ablation mechanism [53,54]: (**a**) schematic illustration of aligned CFs and the carbon matrix in the C/C composites, (**b**) the C/C composites with a single paralleled fiber, (**c**) diagram depicting surface and interfacial oxidation processes, and (**d**) ablation-related SEM images of the three regions.

However, porous C/C composites readily react with oxygen. In addition, various defects and impurities are formed on the surface, and bring about significant mass loss and a rapid decline in mechanical properties at an elevated temperature as active sites owing to the oxidation behavior. Figure 6 shows the morphology and schematic diagram of C/C composites and their ablation process. Paralleled CFs and carbon matrixes within C/C composites (Figure 6a) contain the upper fibers, lower fibers, and carbon matrix. Figure 6b presents the cross-sectional morphology. The fibers aligned parallel to the lamellar planes are progressively burned out or exposed during ablation, resulting in a characteristic “concave” topography. The oxidation process can be kinetically delineated into two distinct regimes: a reaction-controlled regime and a diffusion-controlled one (Figure 6c). When ablating the C/C composites, three distinct zones, named Zones I, II, and III (Figure 6d), appear. Of these, Zone I exhibits a typical pyrolytic carbon (PyC)-coated carbon fiber architecture featuring well-defined ablation pits on drum-shaped fiber bundles. Within a short duration of ablation, numerous ablation pits with a diameter of 5–10 μm are formed on the fiber bundles. At the center of these pits, aligned fiber bundles remain discernible, which is attributed to the markedly lower ablation rate of PyC along the fiber axis direction. Zone 3 resides at the center with the highest temperature. Therefore, the lower parallel fiber bundles present a drum-shaped appearance. As the ablation degree increases, the ablation pits gradually expand and eventually coalesce. By comparing the microstructures of these three zones, the ablation behavior is clearly described [53,54]. Currently, a mass of research focuses on the oxidation mechanism. The oxidation behavior is primarily classified into three types depending on the reaction temperature. Above 800 °C, the ease of oxygen diffusion in the boundary area predominates the oxidation. Between 600 and 800 °C, the degree of oxidation is primarily determined by the oxygen diffusion within the porous structure and material compactness. Below 600 °C, the main reaction takes place between adsorbed oxygen and active sites on the surface.

It is well known that the ablation behavior of the C/C composites comprises two synergistic mechanisms: thermochemical and mechanical ablation. Thermochemical ablation refers to the process of material consumption caused by chemical reactions in oxygen-containing environments at high temperature [55]. Following high-temperature flame ablation, the surface of the C/C composites exhibits three distinct morphologies at central, transitional, and border zones. Ablation initiation typically occurs at microstructural heterogeneities, particularly at the interface between PyC and active points such as crystallographic defects or embedded impurity particles. The orientation of CFs also poses a significant impact on the ablation resistance. Therefore, unveiling the ablation mechanism is significant to understand the structure of TIMs [56].

Alternatively, mechanical ablation includes sheet-like erosion due to high thermal stress damage, and a granular one on the surface due to the strong pressure and shear stress generated by high-speed gas flow [57]. Specifically, the favorable thermophysical properties of the C/C composites enable inward heat conduction during ablation. This establishes a steep thermal gradient across the material thickness, thereby inducing depth-dependent variations in some mechanical parameters such as strength and elastic modulus. Various forms of defects (pores, cracks, etc.) cause stress concentration under the thermal stress. When exceeding the fiber strength, the material begins to flake off from the crack tip. The matrix carbon possesses lower density than the CFs, which causes the matrix to preferentially burn and at a faster rate. As the matrix degenerates and exposes the fibers, the strength of the fibers is greatly reduced. Meanwhile, the fibers undergo rapid amorphization under the intense thermal and mechanical loading imposed by ultra-high-temperature gas flow, followed by delamination and detachment from the matrix. Therefore, mechanical erosion further intensifies the material failure. Note that the C/C composites exhibit a regularly distributed temperature field from inside to outside during ablation, together with inconsistent maximum temperatures and degrees of damage in different areas. Slightly higher and lower morphologies appear at the middle part and periphery after ablation, respectively [58].

The preparation process of the C/C composites is rather complex, and mainly includes three steps of preform molding, preform densification, and graphitization treatment. The content, type and arrangement (orientation) of the fiber, and the internal structure within the CFs, preform in different methods. The matrix carbon, which is mainly used to fix the filling preform and transfer the load, is another important factor affecting the performance. According to the carbon source, the matrix carbon is mainly derived from resin carbon, asphalt carbon, and pyrolytic carbon. Table 3 presents a comparison of the physical properties of typical carbon–carbon composite materials for various matrix carbon molding methods.

**Figure 7:**
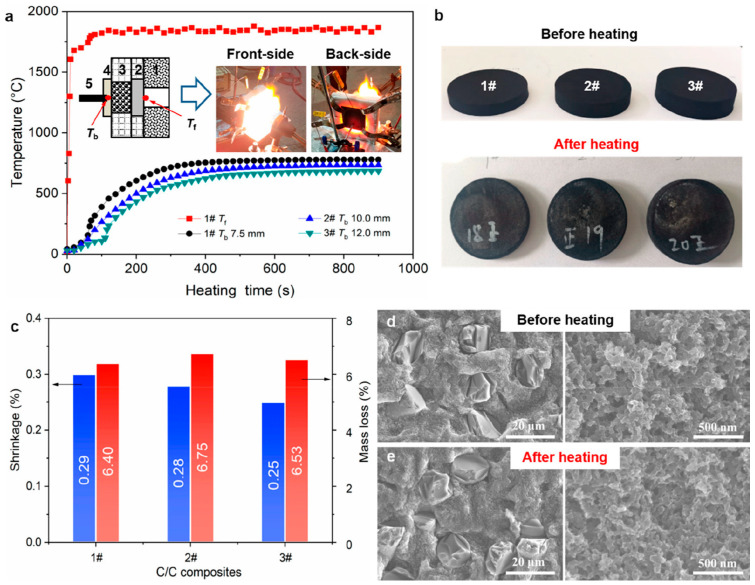
The microstructural evolution of the C/C composites after heating(1#, 2#, 3# represent samples of different thicknesses): (**a**) front- and rear-side temperatures during the flame heating test with oxyacetylene, (**b**) photographs before and after heating, (**c**) dimensional shrinkage and mass loss after heating, and (**d**,**e**) SEM images before and after thermal treatment [62].

Phenolic resin (PR) is the most common precursor in the liquid-phase impregnation of thermosetting resins, which exhibits exceptional ablation resistance and thermal stability particularly in high-enthalpy aerodynamic environments [59]. A three-dimensional cross-linked macromolecule network could be formed through polycondensation. Following that, further high-temperature heat treatment can convert them into carbon materials. Figure 7a shows the C/C composites prepared with PR and hexamethylenetetramine (HMTA) separately serving as a reaction precursor and crosslinking agent [62]. It is found that the C/C composites exhibit outstanding thermal insulation performance—comparable to that of polymer-derived ceramic aerogels at an ultra-high temperature (UHT). This superior behavior is attributed to their nanostructured aerogel-like architecture and low degree of graphitization. Following the heating test, the C/C composites nearly kept their initial disk geometry (Figure 7b), with dimensional shrinkage and mass loss measured at 0.3% and 6.8%, respectively. The related loss rate of thickness attained 0.03 μm s^−1^ (Figure 7c), likely resulting from mild oxidative degradation within the constrained test atmosphere. In addition, both fiber microstructures and the matrix remained intact and morphologically unchanged (Figure 7d,e), confirming exceptional thermo-structural stability at UHT. Note that graphitization is a necessary step after densification as it eliminates defects in the carbon matrix through high temperature, and transforms unstable carbon into stable graphite.

However, the C/C composites maintain the excellent heat resistance only in vacuum or inert atmospheres. The oxidation behavior occurs above 400 °C in air [63], and becomes more severe with temperature. To improve this, the matrix modification technology is feasible [64]. Alternatively, oxygen-barrier coating is capable of achieving the same goal by preventing direct contact between oxygen and the base carbon material [65].

**Figure 8:**
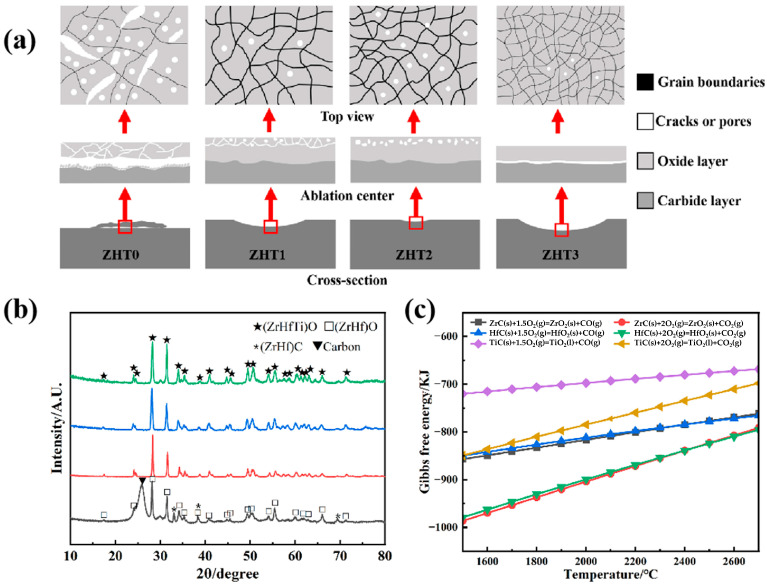
Ablation mechanism and microstructural characterization of (Zr, Hf, Ti)C-modified C/C composites: (**a**) schematic ablation mechanisms for C/C-(Zr, Hf)C and C/C-(Zr, Hf, Ti)C composites, (**b**) XRD patterns of four samples, (**c**) temperature-dependent Gibbs free energy changes (ΔG) for different oxidation reactions [70].

In the aspect of matrix modification, new components, including refractory carbides, silicides and borides of some transition metals (Ti, Hf, Ta, Zr, etc., or various combinations), are always introduced into the carbon matrix. These components possess an elevated melting point (>3000 °C), desirable mechanical properties, and benign endurance to oxidation, thermal shock and ablation. After oxidation, the formed refractory oxides raise the oxidation temperature, while the resultant glassy phase can prevent the inward diffusion of oxygen and enhance the oxidation resistance [66]. Chen et al. [67] develop 3D C/SiC/TaC multilayer composites and find that the best ablation performance is displayed under 2000 °C and 20–40 s flame ablation of an oxygen–acetylene mixture. The mass and linear ablation rates are separately lower than 3.5 mg/s and 25 μm/s. Also, the addition of Ta_0.78_Hf_0.22_C solid solution effectively improves the ablation performance of the C/C composites, and reduces the ablation rate by 73% [68]. Wang et al. [69] prepare ZrC-modified C/C composites. Excellent mechanical erosion resistance is found above 2700 °C. Liu et al. [70] investigate the influence of the Ti dosage on the ablation performance of the C/C–(Zr, Hf, Ti)C composites exposed to an oxyacetylene flame at 2600 °C (Figure 8). The infiltration precursor is prepared by mixing Ti, Zr and Hf powders at varying molar ratios. The composites with elemental molar ratios at 0:67:33, 10:60:30, 20:53:27, and 30:47:23 are labeled as ZHT0, ZHT1, ZHT2, and ZHT3, respectively. Clearly, with an increasing Ti content, the porosity of the resultant oxide layer gradually decreases (Figure 8a), and the protection capability over the underlying ceramic substrate is enhanced. At 30 at% Ti (ZHT3), excess TiO_2_ penetrates the skeleton of (Zr, Hf)O_2_ material, forming the most compact and continuous scale, which corresponds to the lowest mass ablation rate. Thermodynamic analysis shows that the Gibbs free energy to oxidize ZrC and HfC are comparable but much lower than the TiC case within the temperature range of 1800 to 2700 °C (Figure 8c). Consequently, ZrC and HfC are readily oxidized into ZrO_2_ and HfO_2_, and TiC undergoes delayed oxidation into TiO_2_ (Figure 8b).

In terms of coating technology, the used coating isolates the C/C composites from oxygen-rich environments by avoiding the reaction between carbon and oxygen, and extends the service life. Compared with substrate modifications aiming for short-term ablation and medium–low-temperature oxidation resistance, the coatings provide long-term protection toward UHT. As for antioxidation coatings at 1000–2000 °C, glass, metal and ceramics have gained extensive attention. As for ablation-resistant coatings with surface temperatures up to 3000 °C, UHTC is a good choice [71]. Notably, the composites exhibit a compressive strength of 200 MPa, a low CTE (≤6 × 10^−6^/°C), large TC, and exceptional ablation resistance, which render them highly suitable for both fundamental scientific research and demanding engineering applications. However, the C/C composites begin to oxidize at 400 °C, simultaneously releasing CO or CO_2_ gas, and undergo severe ablation and mass loss. Although researchers have developed multilayer coating systems (e.g., SiC/glass–ceramic composites), the mismatch in CTE of the coating and the substrate leads to thermal stress and coating peeling. In addition, the manufacturing process is complex and time-consuming. The fracture toughness of the material remains relatively low, and the interlayer shear strength (only 14–16 MPa) is much lower than that of metallic materials, which are unsuitable for structural applications subjected to complex stresses (especially shear loads).

### 2.3. Ceramic Systems and Their Composites

#### 2.3.1. Common Ceramic Matrix and Its Composites

Ceramic matrix composites (CMCs), e.g., C/SiC ceramics (CFs or SiC fiber-reinforced ceramic matrix), belong to a class of advanced materials owning high temperature resistance and large strength [72]. CMCs possess excellent properties, such as high specific strength, specific modulus, reliability, resistance to temperature and ablation, and low density. Therefore, they show a huge potential to replace metals and their alloys for high-temperature structural materials [73]. Compared with superalloys, CMCs show lower density (about 30% of Ni-based superalloys), higher heat resistance (>1200 °C), and smaller CTE (<9 × 10^−6^/°C for most CMCs).

As for the oxidation mechanism, taking SiC-based CMCs as an example, SiC material possesses passive and active oxidation under low-temperature/oxygen-rich or high-temperature/oxygen-deficient conditions, respectively [74]. Opila et al. [75] explore the impact of the surface SiO_2_ thickness on the oxidation of the C/SiC composites under inert conditions. Due to the manufacturing limitations and differences in thermal properties, a lot of initial cracks and pores within the composites exist. These defects provide oxidation channels into the interior, form an oxidation zone at the crack tip, and eventually result in surface oxidation. Chen et al. [76] employ a high-frequency plasma wind tunnel to generate a highly dissociated atomic-oxygen environment and systematically probe the oxidation of SiC under such conditions. The oxidation of SiC proceeds via atomic oxygen diffusion through structural channels in the SiO_2_ lattice, followed by the reaction with SiC at the SiO_2_/SiC interface to form additional SiO_2_. This process necessitates an activation energy of 60 kJ·mol^−1^. Concurrently, SiO_2_ is lost via sublimation. Under high-temperature and low-pressure conditions, solid SiO_2_ volatilizes to form gaseous SiO_2_, which diffuses through the boundary layer and subsequently re-oxidizes to SiO_2_ in the gas phase or at the surface. This volatilization-driven process results in progressive depletion of the surface SiO_2_ layer, with an experimentally determined activation energy of 115 kJ·mol^−1^. In contrast, under conditions that suppress active oxidation, a protective and adherent SiO_2_ layer is gradually formed on the material surface, effectively mitigating rapid degradation. Moreover, atomic oxygen diffusion in SiO_2_ proceeds analogously to molecular oxygen primarily through three distinct pathways: vacancy-mediated diffusion—dominated by oxygen vacancies, peroxide-assisted diffusion, and interstitial diffusion commonly referred to as channel or tunnel diffusion.

**Figure 9:**
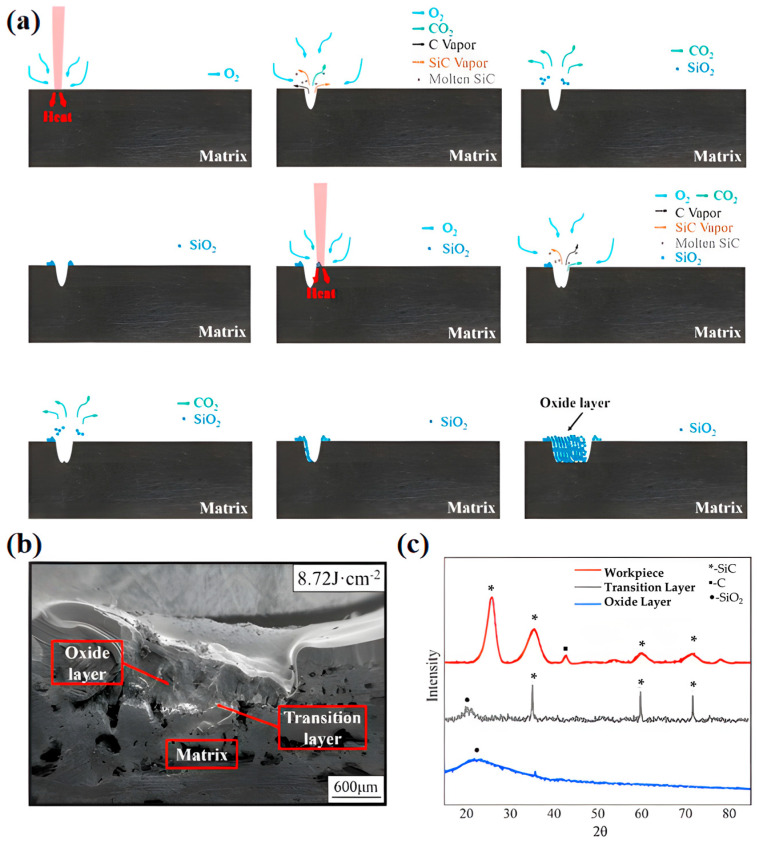
Laser-induced ablative oxidation mechanism of the CFs/SiC composites and characterization of the oxide layer: (**a**) schematic oxidation mechanism, (**b**) cross-sectional morphology of the oxide layer, and (**c**) XRD patterns of the workpiece, transition layer, and oxide layer [77].

In a previous work [77], the morphology and oxidation mechanism of CFs/SiC CMCs are investigated by laser ablation (Figure 9). The workpiece rapidly absorbs energy upon laser irradiation within an extremely short duration (Figure 9a). Then, the temperature quickly rises in the local region, which melts and vaporizes SiC. A portion of the CFs is gasified, while another fraction reacts with ambient oxygen to form CO_2_. The phase transitions and gaseous reaction products lead to a sudden volumetric expansion. Combined with the shockwave and explosive effects of the high-energy laser pulse, the material is ejected and the gases are diffused into surrounding atmospheres. The molten materials and vapor are fully exposed to ambient oxygen, leading to the formation of SiO_2_ and CO_2_. Finally, CO_2_ gas is released into the environment, while solid SiO_2_ is deposited on both sides of the laser beam path. With successive scanning cycles, oxide material gradually builds up in the kerf, eventually forming a loose and porous oxide layer. Meanwhile, numerous pores are developed within the oxide layer. When the laser energy density attains 8.72 J·cm^−2^, the oxide layer reaches a thickness of around 850 μm (Figure 9b). Under these conditions, the layer exhibits a highly porous and loosely packed morphology. From XRD patterns (Figure 9c), it is found that SiO_2_ serves as the primary crystalline phase in the oxide layer. During laser irradiation, CFs and SiC react with ambient oxygen, which generates gaseous species of CO and CO_2_. Moreover, SiO_2_ as a solid reaction product is deposited on the surface of the workpiece.

CMCs can be classified into oxides [78] and non-oxides based on the composition or ceramic matrix. Non-oxide ceramics possess the advantages of a low CTE, desirable TC, high strength at elevated temperatures, and good resistance to thermal shock and corrosion. Thus, they are extensively employed in the aspects of metallurgy, electronics, chemical engineering and machinery. Non-oxide CMCs mainly include SiC, Si_3_N_4_, and the emerging SiBCN [79]. Song et al. [80] prepared porous and multifunctional SiC matrix composites via CVD and simple heat treatment. The formed dense SiO_2_ possesses a very low oxygen permeability at 1200 °C. Hu et al. [81] investigated the SiC ceramic matrix composite phase-change materials (PCMs). The main strategy is to encapsulate PCMs of high latent heat with porous SiC ceramics featuring a low TC of 0.402 W/(m·K) and high-temperature resistance, which mitigates the high-temperature impact in aerothermal environments. This behavior is attributed to the high enthalpy of the PCMs, which enables substantial thermal energy storage or release during melting or solidification while holding the system temperature nearly constant near the phase transition temperature. In previous work [82], Zr-modified SiC-based nanofiber aerogels (ZSNFAs) with hierarchical microarchitectures are prepared through a combined electrospinning and carbothermal reduction process. The resulting ZSNFAs exhibit a compressive strength of approximately 0.32 MPa, and ultra-low TC of 0.023–0.024 W/(m·K), rendering them highly suitable for thermal insulation in high-temperature applications. In contrast, conventional non-oxide CMCs are susceptible to oxidative degradation at elevated temperatures. Therefore, their applications in high-temperature and oxygen-rich environments are largely limited [83].

Oxide CMCs mean that the matrix and reinforcing components are both oxides. The inherent oxidation resistance, toughness, specific strength and specific modulus can effectively overcome the sensitivity to cracking and thermal shock, and bring about a longer service life in high-temperature aerobic environments. Meanwhile, the abundant raw materials and low production cost can significantly reduce the application cost. Currently, the common oxide matrices include quartz, Al_2_O_3_ [84], MgO, Mullite, ZrO_2_, yttrium aluminum garnet (YAG), lithium aluminum silicon (LAS), barium aluminum silicon (BAS), etc. The performance of some oxide matrices is shown in Table 4.

Among them, Al_2_O_3_ is currently the most popular matrix material. Reduless-Wrenn et al. [89] prepared Al_2_O_3_ matrix composites with excellent mechanical properties via ATK-COI ceramics. However, Al_2_O_3_ ceramics are prone to creep failure at high temperature. Xu et al. [90] investigated SiO_2_ ceramic/aerogel (QF-SA) composites fabricated from a three-layer graded quartz fiber felt. Here, in situ pyrolysis of a silica sol precursor impregnated within the thermal protection substrate is adopted to prepare the SiO_2_ ceramic layer, whereas a facile sol–gel method is employed to synthesize the insulating layer. The intermediate layer consisted primarily of SiO_2_ and SiO_2_-based aerogel. Under irradiation from a quartz lamp, the composites exhibited a surface temperature that stabilized at 800 °C following an initial transient peak of 1000 °C. For the QF-SA1 variant, the backing plate temperature reached a maximum of 232 °C after 1000 s. Its TC ranging from 0.022 to 0.042 W/(m·K) could provide effective thermal insulation, as evidenced by outstanding thermal protection performance in high-temperature testing.

Although ceramic materials possess high hardness, excellent thermal stability and chemical stability, they still suffer from high brittleness and low fracture toughness. To enhance the toughness, a second phase is introduced. Toughening mainly relies on the size effect to reduce defects, and the composite effect to increase crack propagation resistance.

**Figure 10:**
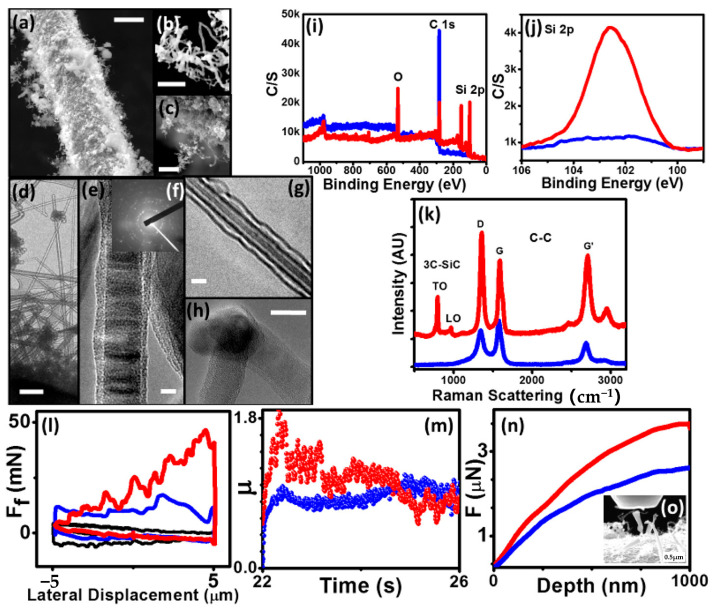
Structural and mechanical characterization of SiCNT/NW-SiCF composites: (**a**–**c**) SEM images at increasing magnifications: (**a**) 10 μm, (**b**) 0.5 μm, and (**c**) 2.5 μm, (**d**–**h**) TEM images with diffraction pattern (**f**) and with scale bar: (**d**) 5 μm, (**e**) 10 μm, (**g**) and (**h**) 20 nm. (**i**) XPS survey spectra of CNT–SiCF and SiCNT/NW-SiCF, (**j**) high-resolution XPS spectra of the Si 2p region, (**k**) Raman of SiCNT/NW-SiCF and CNT-SiCF samples, (**l**) lateral force–displacement curves measured via nanoscale friction testing: naked SiCF (black), CNT-SiCF (blue), and SiCNT/NW-SiCF (red), (**m**) the friction coefficient versus time plots under constant normal load, compression load–displacement curves: (**n**) CNT–SiCF and (**o**) SiCNT/NW-SiCF, inset shows the corresponding SEM image of the indented region post-test [91].

The current reinforcing phases mainly include traditional particles, whiskers and fibers, as well as emerging low-dimensional nanomaterials such as CNTs [91] and graphene. For instance, the transformed SiCNT/nanowires (NWs) imitate the morphology of CNT on the SiC fiber (SiCF), forming an integrated fibrous network (Figure 10a–c). TEM images (Figure 10d–h) reveal a well-distributed architecture comprising straighter MWs and more curved nanotubes, along with clear interfacial junctions. XPS analysis (Figure 10i) demonstrates that the carbon peak is higher and the Si 2p peak is lower for the CNT–SiCF compared to the SiCNT/NW–SiCF (Figure 10j). No SiC peak and a low D-to-G intensity ratio (I_D_/I_G_) in the Raman spectra (Figure 10k) collectively confirm the exclusive presence of well-ordered CNTs on the SiC fibers. Following a 5 h transformation, high SiC peaks and a substantially increased I_D_/I_G_ appear, corroborating the structural conversion of CNTs into SiC. To evaluate the interfacial integration of SiCNT/NWs within the SiC fiber matrix, the fuzzy fiber was subjected to lateral friction and nanoscale compression tests analogous to those performed on CNT–SiCF. The lateral force is required to displace a probe across the SiCNT/NW surface, which is comparable to that measured for pristine CNTs and bare SiC fibers (Figure 10l). This indicates full embedding of the SiCNT/NWs within the SiC fiber substrate. The intimate interfacial contact between the SiCNT/NWs and the underlying SiC fiber results in a coefficient of friction for SiCNT/NW–SiCF analogous to the CNT–SiCF counterpart (Figure 10m). The load–displacement curves (Figure 10n,o) show that SiCNT/NW–SiCF sustains significantly higher applied loads at equivalent indentation depths compared with CNT–SiCF, indicating a superior load-bearing capacity and structural integrity.

Fiber-reinforced composites are essentially axial particles embedded in the matrix with high strength and a high elastic modulus. CFs are cheap and show a strong performance under UHT. For example, Kumar et al. [92] prepared CF/SiC CMCs using the precursor cracking method. Due to the “pull-out effect” of CFs, the fracture toughness and flexural modulus reached 14.5 MPa and 73 GPa, respectively. Ma et al. [93] prepared high-performance CF-reinforced CMCs with low cost and high efficiency through in situ hot-pressing densification, together with a compressive strength of 308.51 MPa and flexural strength of 115.68 MPa. Compared with CFs, SiC fiber possesses a higher melting point of 200 °C. Kang et al. [94] successfully prepared SiC fibers with an average diameter of approximately 700 nm by using electrospinning and high-temperature sintering techniques. The fibers kept stable to 1800 °C and had good oxidation resistance at 1400 °C and a high-temperature thermal insulation of 0.273 W/(m·K) at 1200 °C, but showed inferior mechanical properties and modification effects.

Most oxide-reinforced fibers are polycrystalline ceramic fibers, mainly composed of Al_2_O_3_, Al_2_O_3_-SiO_2_ ceramics and substances such as B_2_O_3_, ZrO_2_, MgO, etc. They show a high tensile strength and modulus, and excellent resistance to high-temperature oxidation. For example, Al_2_O_3_ fiber is a new type of high-performance inorganic fiber with Al_2_O_3_ as the main component (>60%) and a few SiO_2_, Fe_2_O_3_, B_2_O_3_, ZrO_2_, Y_2_O_3_ or more as secondary components. Al_2_O_3_ fibers show excellent mechanical and thermophysical characteristics, such as a large tensile strength and elastic modulus, low density, low TC, and a low CTE. Compared with non-oxide counterparts, Al_2_O_3_ fibers also possess favorable resistance to high-temperature oxidation [95]. At present, commercial oxide fibers mainly include 3M’s Nextel series fibers, Dupont’s FP, PRD-166 series fibers, etc. Most oxide fibers are polycrystalline with many slip planes, and are prone to creep and grain growth during long service at a high temperature. Among these, 3M’s Nextel 720 (3M Company, St. Paul, MN, USA) fiber is a composite microstructure comprising mullite and Al_2_O_3_ phases. It possesses outstanding resistance to creep deformation and can retain approximately 86% of its room-temperature strength after exposure to 1400 °C. These merits make it a globally favored reinforcing fiber for the development of advanced oxide-based CMCs [96].

Ceramics and their composites are resistant to high temperature, oxidation, and ablation, and possess higher strength, lower density and stronger oxidation resistance than metallic materials, C/C and their composites. Thus, they are widely used in high-temperature environments typically ≤1600 °C (oxide-based) or ≤1400–1650 °C (SiC-based). Despite the toughening of ceramic substrates with reinforcing materials, their overall toughness is still weaker than that of metallic materials. Further research should be carried out to enhance the toughness and increase the upper limit of resistance to high temperature and oxidation.

#### 2.3.2. UHTCs and Their Composites

At present, superalloys show relatively weak oxidation resistance with operating temperatures below 1200 °C [97]. Moreover, the C/SiC and C/C composites are extensively employed in high-temperature scenarios to 1600 °C, owing to their superior mechanical performance and resistance to oxidative degradation under elevated thermal conditions. At higher temperature (above 2000 °C), however, these composites show reduced oxidation resistance and structural stability, and are readily ablated with material loss, thus being unsuitable for more demanding conditions [98,99]. Based on these characteristics, UHTCs can be used as an alternative to high-temperature TIMs [100].

UHTCs belong to refractory materials characteristic of exceptional intrinsic thermal stability, primarily comprising transition-metal carbides, nitrides and borides [101], especially those of Group IV and V elements, such as Ti, Zr, Nb, Hf, and Ta [102]. UHTCs generally possess a high melting point above 3000 °C [103], and can operate at temperature up to 2000 °C and above [104]. Moreover, UHTCs exhibit a combination of exceptional properties in the aspects of thermal stability, mechanical strength and hardness, resistance to oxidation, elastic modulus, wear resistance, TC and electrical conductivity, and creep resistance. The most significant difference from CMCs is that the upper limit of high temperature is greatly enhanced up to 3000 °C, while maintaining excellent resistance to oxidation and ablation [105]. This behavior arises from the exceptionally strong covalent and metallic bonding (present in ceramic borides, carbides, and nitrides), and bonding configurations that confer outstanding structural stability under extreme thermal and mechanical loadings.

The representative UHTCs and their composites include ZrB_2_ [106], HfB_2_, ZrC, HfC [107], HfB_2_-SiC [108], ZrB_2_-SiC [109], ZrC-TiC, etc. Of these, nitride-, boride- and carbide-based ceramics possess melting points of 1900–3000 °C, 2500–3250 °C, and 3000–3890 °C, respectively. Boride ceramics could be oxidized to B_2_O_3_, which becomes liquid at a relatively low temperature [110]. Nevertheless, at temperatures ≤ 1000 °C, the formation of a protective amorphous or liquid B_2_O_3_ layer affords effective oxidation resistance to the underlying boride phase. At a higher temperature, the liquid evaporates and the protection mechanism fails. The eutectic temperature of Group IV carbides with C/C substrates is surprisingly high (2910 and 3180 °C for ZrC and HfC) [111]. Interstitial carbides (Hf, Zr, Ti, Ta, etc.) form the largest class of carbides, and possess one of the highest melting points and high strength at elevated temperature due to the strong carbon network. However, they are less resistant to oxidation than borides [112]. The gases released during the oxidation process create a non-protective oxide layer at temperatures up to 1500 °C, which causes severe flaking. Nitrides show nearly the same properties as carbides, but are less studied for Group IV UHTCs. Despite a relatively high melting point, their oxidation protection performance is as poor as that of carbides since the oxidation products do not exhibit passivation effects [113].

The resistance to oxidation and ablation of UHTCs are predominantly determined by the chemical composition and microstructural architecture of the surface oxide layer. Adding SiC to the matrix helps to form glassy SiO_2_, which improves the ablation resistance [114]. The insufficient material densification during the ablation process could produce micropores, which serve as channels for oxidizing the gas into the composites [115]. As for Zr-based materials [116], ZrC and SiC are firstly oxidized into SiO_2_ and ZrO_2_ and then melt. The oxidation destroys the fiber structure of the composites, and makes the oxide layer loose and porous. Eventually, under the action of pneumatic pressure, the surface oxide layer peels off, accompanied by the evaporation of SiO_2_ and the release of SiO and CO gases. Zr-based UHTCs deliver exceptional resistance to ablation and oxidation mainly due to the resultant formation of a dense ZrO_2_ layer on the surface. This layer can block the diffusion of ambient oxygen molecules. Therefore, more volume fraction of ZrB_2_ and ZrC within the matrix will produce more ZrO_2_ during use, which significantly improves the resistance to ablation and oxidation.

**Figure 11:**
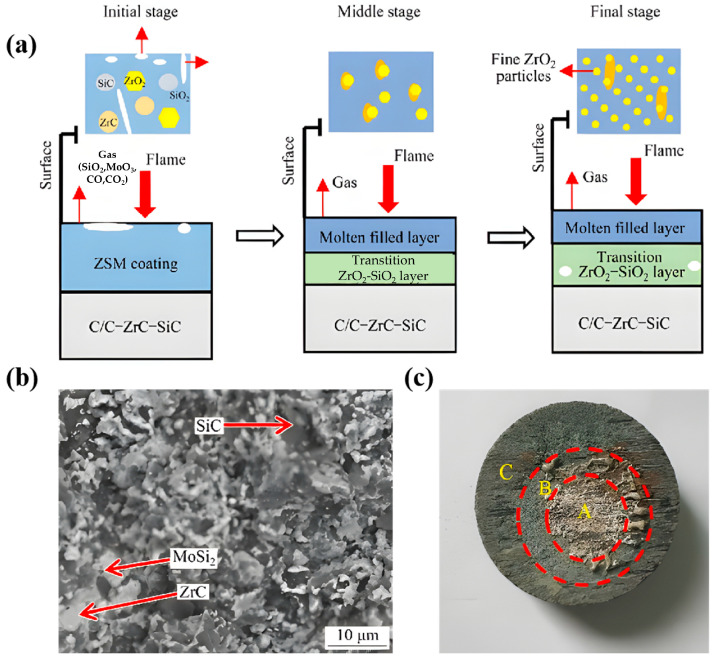
The ablation mechanism and morphologies before and after ablation of ZrC-SiC-MoSi_2_ ceramics: (**a**) schematic diagram of coating ablation mechanism, (**b**) SEM of samples, (**c**) samples after 60 s of ablation [117].

Previous studies [117] have witnessed that the ablation of ZrC-SiC-MoSi_2_ coatings is closely related to multiple forms of chemical erosion and mechanical degradation (Figure 11a). Chemical erosion arises predominantly from high-temperature oxidation between the coating constituents (ZrC, SiC, and MoSi_2_) and atmospheric oxygen. Mechanical degradation encompasses thermal stress-induced cracking, localized melting, and the erosive removal of surface material driven by rapid gas evolution including CO, CO_2_, MoO_3_, and silicon-bearing species (e.g., SiO and SiO_2_) under a combustion gas flow with high velocity and pressure. The ablation behavior of the coatings proceeds through three distinct stages. Initially, ZrC, SiC, and MoSi_2_ are oxidized into solid ZrO_2_ particles, molten SiO_2_, and gaseous MoO_3_, respectively. In this case, the coating serves as a protective barrier by consuming substantial amounts of oxygen, and thus minimizes the oxidative eroding of the underlying substrate. As a result, the oxidation rate of ZrC and SiC primarily determines the ablation rate. In the intermediate stage, SiO_2_ reacts with ZrO_2_ and produces molten zircon (ZrSiO_4_), which infiltrates the porous ZrO_2_ matrix with excess SiO_2_, and effectively fills voids to significantly reduce the porosity. Under these conditions, the formation rate of the liquid phase exceeds its removal rate via evaporation and high-velocity gas flow. Consequently, the oxygen diffusion velocity through the oxide layer governs the ablation rate in this stage. Finally, extensive SiO_2_ evaporation produces numerous pores within the oxide scale, and results in a porous and loose ZrO_2_ skeleton on the surface. Under these conditions, the evaporation rate of the liquid phase exceeds its filling rate into defects, meaning the overall ablation kinetics are limited by oxygen diffusion through the underlying bulk material. The pre-ablated coating exhibits a dense and continuous microstructure (Figure 11b) with MoSi_2_ uniformly distributed around ZrC grains, thus effectively filling intergranular spaces. In addition, the superior wettability and ductility of MoSi_2_-decorated ZrC promote efficient sintering and enhance the densification of the ZrC–SiC–MoSi_2_ composites. Three distinct surface regions are clearly identifiable on the ablated specimen: central (A), transitional (B), and boundary (C) zones (Figure 11c). The central region is subjected to the most intense thermal loading and mechanical erosion, where a dense oxide layer was generated. Compared with common CMCs, UHTCs exhibit certain differences in their oxidation mechanisms and failure modes, as shown in Table 5.

However, traditional UHTCs such as brittle HfC and ZrC show poor resistance to thermal shock despite their high melting points, which limits their practical applications. To overcome these problems, new materials such as high-entropy UHTCs (HE-UHTCs) have emerged, which comprise carbides, borides, or nitrides of various metal elements [118]. Peng et al. [119] prepared high-entropy ceramics using ten transition metal carbide and nitride ceramic powders, and estimated the ablation features at 2227 °C. It was found that the mass ablation rate and linear ablation rate were reduced by 57 ± 1% and 72 ± 1%, respectively, via nitrogen incorporation. Moreover, lattice distortion-induced phonon scattering in HE-UHTCs leads to reduced TC with respect to their single-component counterparts, thereby enhancing the thermal insulation capability.

HE-UHTCs have become important research targets in the field of UHTCs. The fracture toughness has been improved to some extent by adding sheet-, granular- or whisker-like reinforcing materials. Currently, the main toughening methods resemble those for common CMCs, including toughening with particles, soft phases, short fibers and continuous fibers [120]. For example, the incorporation of SiC particles into a ZrB_2_ matrix (ZrB_2_–20 vol% SiC) enhances the fracture toughness to 7.8 MPa·m^1/2^ and the flexural strength to 549.8 MPa, demonstrating a pronounced toughening effect [121]. Also, the toughness of the ceramic material could be increased by incorporating a soft phase with low hardness and a low elastic modulus to prevent crack propagation. Cheng et al. [122] used chopped PyC-coated CFs to enhance ZrC-SiC, resolving the contradiction between strength and toughness. Studies have shown that Hf and Zr diborides containing continuous CF reinforcing phases remain stable at temperatures above 2500 °C in UHTCs [123].

UHTCs generally possess a melting point above 3000 °C, and maintain high strength in an inert environment at 2000–3000 °C, far exceeding traditional alloys and common ceramics (such as SiC-based CMCs ≤ 1650 °C). Compared with CMCs, UHTCs show higher resistance to oxidation and ablation. However, UHTCs are brittle and prone to sudden fracture under an impact load. The relevant manufacturing is difficult and costly. Moreover, difficult densification, a low yield, expensive raw materials (Hf, Ta, etc.), and the introduction of some metallic materials lead to an increased density.

## 3. Conclusions

As the Mach number of high-speed aircraft increases, the thermal barrier encountered by thermal protection systems during service becomes particularly prominent. The surfaces of thermal protection structures, including the nose cone, leading edge, combustion chamber and nozzle of the scramjet engine, need to withstand the aerothermal effect of high heat flow and pressure. In addition, the surface temperature can reach over 2000 °C in a short period of time, which puts more requirements on the high-temperature mechanics, thermal shock, and oxidation resistance of TIMs. To further enhance the payload capacity, research on the comprehensive properties of TIMs is also required, including the endurance of a high temperature, ablation and oxidation, toughening, light weight, resistance to thermal shock and wear, etc. Table 6 concludes the feature properties, main advantages and current challenges of various thermal protection material systems.

(1) Metal-based and -doped composites exhibit high toughness, high melting points, resistance to thermal shock and wear, easy processing, etc. Their working temperature is generally around 1000 °C. Currently, the most studied application is Ni-based superalloys, which can reach 1200 °C. However, these materials are readily oxidized, and the oxides are prone to shedding and volatilization in high-temperature environments. To enhance the hardness and oxidation resistance, HEAs systems are developed. Due to slow diffusion, they delay the combination of ionic metal and oxygen species, and they promote reactions between certain volatile and simple oxides to produce protective layers. However, their inherent large density seriously affects the effective payload of aircraft and restricts further development.

(2) The biggest difference between C/C and its composites and metallic materials is the low density, which is beneficial for a significant weight reduction. In addition, the C/C-based composites exhibit extremely strong heat resistance. A high specific strength and good fracture toughness are maintained at a high temperature, especially with the increased strength with temperature (1000–2300 °C). In addition, the C/C-based composites remain stable above 3000 °C and can withstand temperatures of 3800–4000 °C in inert gas atmospheres. However, poor oxidation resistance above 400 °C and brittleness exist. Despite CF toughening, the toughness is still inferior to that of superalloys, and a high preparation cost and complex technical process restrict the further progress.

(3) Although CMCs possess excellent properties in the field of high-speed aircrafts, UHTCs bear a much higher upper limit of heat resistance while maintaining excellent resistance to oxidation and ablation. Both exhibit lower density compared to superalloys. However, ceramic materials have relatively high brittleness and poor thermal shock resistance. To overcome these issues, the high-entropy effect could be leveraged to prepare HE-UHTCs.

With the ever-increasing development of aerospace technology, the higher flight speeds, more frequent extreme environments, larger loads and favorable structural efficiency for the thermal protection systems have posed thorny challenges to resistance to the oxidation and ablation as well as mechanical properties of TIMs. Despite the existing progress, more attention should be paid in the following aspects.

(1) It is necessary to continuously overcome the shortcomings of different material systems and raise the temperature limit. As for metal-based materials, it is necessary to suppress high-temperature oxidation volatilization through composition optimization and introduce rare earth elements into RHEAs to form stable oxide layers. As for C/C composites, gradient coatings with both long-term oxidation resistance and toughness should be developed, in combination with substrate doping technology to achieve a wide temperature range for protection. The brittleness bottleneck needs to be broken through for UHTCs while nano-reinforcing phases or grain boundary engineering can be used to enhance the thermal shock resistance.

(2) Multiscale co-design of composite systems. It is indispensable to consider the composite modification of multiple materials to integrate the advantages of each component. High temperature resistance, low density and good interface compatibility should be considered. Moreover, using UHTCs as coatings for the C/C composites is feasible to reduce the oxidation possibility of the internal materials.

(3) Green and efficient innovation in the preparation process. More efforts should be paid to ensuring a low cost and further developing new technologies, such as microwave sintering and laser additive manufacturing, to shorten the preparation cycle of the C/C composites.

## Figures and Tables

**Table 1:** Comparison table of typical aircraft speeds.

Aircraft/Missile	Type	Maximum Instantaneous Speed (Ma)	Stable Operating Speed (Ma)	Ref.
NASA X-43	Unmanned test aircraft	9.8	6–7	[1]
German “SHEFEX”	Re-entry aircraft	-	6–11	[2]
China’s DF-17	Hypersonic missile	10	6–8	[2]
Australian HIFIRE-7	Free-flying scramjet	8	6	[3]
American X-51A Waverider	Unmanned test aircraft	5.1	5.1	[4]

**Table 2:** Comparison of key properties for superalloys, refractory alloys, and high-entropy alloys.

Material Category	Typical Representative	Melting Point	Operating Temperature	Oxidation Resistance	Ref.
Superalloy	Ni-based	~1200 °C	~1000 °C	Poor	[28]
Refractory Alloy	Co-Re-based	~1400 °C	1000~1300 °C	Poor	[36]
High-Entropy Alloy	Hf-Nb-Ta-Ti-Zr	>2000 °C	<1300 °C	Good	[42]

**Table 3:** Comparison of key properties for different carbon matrix types.

Matrix Carbon Type	Typical Precursor	Density (g/cm^3^)	Mechanical Properties	Temperature Limit (°C)	Ref.
Resin-derived Carbon	Phenolic Resin (PR)	0.18–0.32	Tensile Strength: ~230 MPa	>2500	[59]
Pitch-derived Carbon	Petroleum Pitch	~2.02	Flexural Strength: 70.3 MPa Compressive Strength: 123.3 MPa	>2200	[60]
Pyrolytic Carbon	Ethanol, Methane, Natural Gas	1.67–1.79	Flexural Strength: 118.9–220 MPa	>2000	[61]

**Table 4:** Properties of common oxide substrates.

Ceramic Matrix	Density (g·cm^−3^)	Melting Point (°C)	Coefficient of Thermal Expansion (10^−6^·°C ^−1^)	Elasticity Modulus (GPa)	Ref.
Al_2_O_3_	3.95	2050	8.4	375	[85]
ZrO_2_	5.68	2715	7.7	169	[86]
YAG	4.60	1970	8~9	-	[87]
Mullite	3.23	1828	5.5	417	[88]
MgO	3.58	2852	13.5~15.0	-	[88]
Quartz	2.3~2.65	1700–1750	-	-	[88]

**Table 5:** Oxidation mechanisms and failure modes of typical CMCs and UHTCs.

	Typical System	Oxidation Mechanism	Primary Failure Cause	Ref.
CMCs	SiC/SiC	Formation of a protective SiO_2_ layer upon oxidation	Interfacial debonding, matrix microcrack propagation	[74]
	Al_2_O_3_/Al_2_O_3_	Intrinsic oxidation resistance	High-temperature creep, fiber/matrix debonding	[84]
UHTCs	ZrB_2_-SiC	Multi-phase oxidation with glassy-layer formation	Pore formation in the SiC-depleted layer, thermal shock cracking	[106]
	ZrC-SiC	Formation of a metal oxycarbide (MeC_x_O_y_) transition layer	Grain boundary softening, volatilization of the oxide layer	[116]

**Table 6:** Comparison of performance of various material systems.

		Operating Temperature (°C)	Resistance to Oxidation	Density (g/cm^3^)	Coefficient of Thermal Expansion (/°C)	Mechanical Property	Major Advantages	Limitations	Ref.
Metal-based alloys		600–1200	Poor	High (Ni-based: >8.5)	Al-based: 7.5 × 10^−6^–12 × 10^−6^	Flexure strength: >400 MPa Modulus: >100 GPa	Good toughness and fatigue resistance	Insufficient antioxidant capacity, high density	[30]
C/C composites		>2200	Extremely poor (<400 °C)	Minimum (0.18–2.2)	0.6 × 10^−6^–1.4 × 10^−6^	Compressive strength: >80 MPa Modulus: >50 GPa	High temperature resistance, wear resistance, low density	Very prone to oxidation	[51]
Ceramic systems	CMCs	<1650	Excellent	Lower (Al_2_O_3_ ceramic: ~3.2 SiC ceramic: ~3.05)	4.0 × 10^−6^–15.0 × 10^−6^ (Al_2_O_3_ ceramics: ~8.4 × 10^−6^) <9 × 10^−6^ (ZrC ceramic: 6.2 × 10^−6^–8.1 × 10^−6^)	Flexure strength: >100 MPa (ZrB_2_ ceramic: 275–480 MPa) Modulus: >100 GPa (ZrB_2_ ceramic: ~489 GPa)	Resistant to high temperatures and oxidation	High brittleness, low fracture toughness, and complex preparation process	[84]
	UHTCs	>2000		Higher (ZrC ceramic: ~6.64)					[101]

## Data Availability

No new data were created or analyzed in this study. Data sharing is not applicable to this article.
